# Critical heat flux enhancement in pool boiling through increased rewetting on nanopillar array surfaces

**DOI:** 10.1038/s41598-018-22693-z

**Published:** 2018-03-19

**Authors:** Thien-Binh Nguyen, Dongdong Liu, Md Imrul Kayes, Baomin Wang, Nabeel Rashin, Paul W. Leu, Tuan Tran

**Affiliations:** 1 School of Mechanical and Aerospace Engineering, Nanyang Technological University, 50 Nanyang Avenue, Singapore, 639798 Singapore; 2 Department of Industrial Engineering and Department of Mechanical Engineering and Materials Science, University of Pittsburgh, Pittsburgh, 15261 USA

## Abstract

Boiling is a key heat transfer process for a variety of power generation and thermal management technologies. We show that nanopillar arrays fabricated on a substrate enhance both the critical heat flux (CHF) and the critical temperature at CHF of the substrate and thus, effectively increase the limit of boiling before the boiling crisis is triggered. We reveal that the enhancement in both the CHF and the critical temperature results from an intensified rewetting process which increases with the height of nanopillars. We develop a predictive model based on experimental measurements of rewetting velocity to predict the enhancement in CHF and critical temperature of the nanopillar substrates. This model is critical for understanding how to control boiling enhancement and designing various nanostructured surfaces into specific applications.

## Introduction

Boiling is a central phenomenon in technological and industrial applications as diverse as thermal management in electronics, power generation and chemical processing^1–3^. In such applications, it is of immense importance to enhance the energy efficiency by increasing the critical heat flux (CHF), the highest heat flux a boiling substrate can achieve, as well as reducing operational risks caused by the notorious “boiling crisis”, a catastrophic failure in boilers or heat exchanging devices^4^. The boiling crisis occurs on a boiling substrate when excessive vaporisation of liquid forms a vapour layer that severely impedes heat transfer through the substrate. This leads to abrupt jump in surface temperature and subsequently irreversible damages to the substrate of the boiling equipment. The temperature *T*_*c*_ at which CHF occurs therefore is directly connected to the boiling crisis; a boiling system operating at temperature higher than *T*_*c*_ inevitably drifts towards the boiling crisis. As a result, an enhancement in boiling performance, without triggering the boiling crisis, requires significant increases in both the critical heat flux and critical temperature *T*_*c*_.

A large body of research has been dedicated to understanding the mechanism of nucleate boiling, a boiling regime in which latent heat transfer is dominant compared to convective heat transfer. With increasing surface temperature, CHF is the upper bound of heat flux in the nucleate boiling regime before the boiling crisis occurs^5^. Various microscale models have been proposed to capture nucleate boiling heat transfer mechanisms^6^. In addition, numerous key parameters affecting the heat flux in the nucleate boiling regime have been identified, e.g., surface roughness ranging from nano- to micro-scales^7^, and wettability^8^. Nonetheless, a robust mechanistic prediction of nucleate boiling heat transfer for a wide range of boiling conditions, including the surface properties, is still not available^9^.

To increase the critical heat flux, numerous methods have been explored following two major approaches: fluid modification and surface treatment. The former approach, which uses additives^10,11^ or nanoparticles^12^, has not been widely applicable as it puts constraints on fluid selection and operating conditions of the boiling system^13^. The latter approach includes either treatments to enhance surface wettability^14,15^, or surface morphological alteration with porous coatings^16,17^, artificial fins^18–20^ and nano/microstructures^13,21^. Although numerous surface modification methods have been found to increase CHF, in particular those utilising nano/microstructures, the roles of surface structures at different length scales in changing the heat flux and the critical temperature remain elusive. On one hand, it was shown that the wickability effect is the major cause of enhancement in CHF^13,21^, i.e., the CHF value was shown to increase linearly with the wicked volume flux for various substrate materials and different types of structures ranging from nanoscales to microscales^21^. On the other hand, the separate effect of variation in nanoscale structures on CHF and the critical temperature is still a standing question. Although it has been suggested that nanoscale structures may intensify the wetting velocity, which is a crucial factor leading to CHF enhancement^15,22^, the relation between a systematic change in nanoscale structures, wetting velocity and CHF has not been established. Moreover, little effort has been made to relate surface wettability or nanoscale structures to the change in the critical temperature *T*_*c*_.

In this study, we investigate the boiling phenomenon of FC-72 on nanostructured surfaces. Fluorinert liquid FC-72 is an ideal coolant liquid commonly used in various heat management applications owning to its low boiling point at 56 °C. We show that modifying the surface morphology at nanoscales leads to significant enhancement in the boiling performance, including both the critical heat flux and the critical temperature. By using nanopillars with systematically varying heights on silicon substrates, we demonstrate scalable boiling enhancement with increasing height of nanopillars. We propose and experimentally verify a mechanistic and predictive model relating the nanopillar-induced increase in rewetting to the enhancement in both CHF and critical temperature.

## Results and Discussion

### Enhancement in critical heat flux and nucleate boiling limit

**Figure 1:**
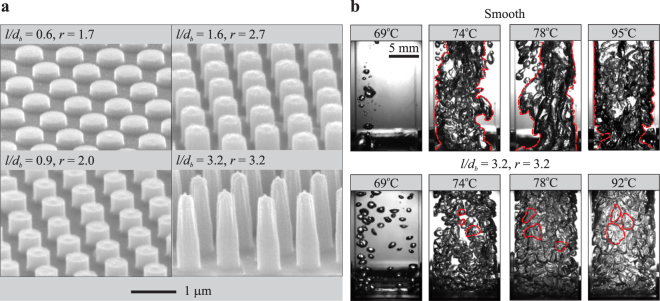
SEM images showing nanopillar substrates of different pillar dimensions: the ratio *l*/*d*_*b*_, defined as the ratio between the pillar height *l* and base diameter *d*_*b*_, and the corresponding surface roughness *r* are indicated in the labels. (**b**) Representative snapshots showing boiling phenomenon on the smooth substrate (top panel) and the nanopillar substrate having *r* = 3.2 (bottom panel) at several surface temperatures *T*. While the smooth surface produces vapour bubbles which have tendency to coalesce (e.g., at *T* = 74 °C) and form vapour columns (e.g., at *T* = 78 °C), the nanopillar substrate produce bubbles which have a narrow size distribution tend to evolve separately without merging. The vapour column and bubbles are highlighted by red dashed lines.

We systematically vary the height *l* of nanopillars fabricated on silicon substrates (Fig. 1a) and show that increasing *l* leads to enhancement in both the critical heat flux (CHF) and the upper limiting temperature of nucleating boiling. The nanopillars are fabricated by an inductively coupled plasma reactive ion etching (ICPRIE) process that directly modifies the surface without adding coating layers that may induce additional thermal resistance to the substrate^23^. Typically, the quantity used for characterising the upper limiting temperature is either the CHF temperature *T*_*c*_, or the Leidenfrost temperature *T*_*L*_. The nanopillars with the base diameter *d*_*b*_ ≈ 440 nm are arranged in a hexagonal lattice with the lattice pitch *p* ≈ 800 nm. The pillar height *l* is varied from 260 nm to 1390 nm. The differences in boiling behaviours and performances between these substrates therefore are attributed to variation in *l*, or alternatively the surface roughness *r* = *A*_*t*_/*A*_*p*_. Here *A*_*t*_ is the total surface area due to the presence of the nanopillars, and *A*_*p*_ is the projected boiling area. In our experiment, *r* ranges from 1 for the smooth silicon substrate to 3.2 for the nanopillar substrate with *l* = 1390 nm.

To qualitatively evaluate the effect of the nanopillars on boiling, we first compare the boiling behaviours between the smooth substrate and the nanopillar substrate with *r* = 3.2 at several surface temperatures *T* (Fig. 1b). Although the onset of boiling occurs at *T* ≈ 69 °C for both substrates, the size of bubbles generated on the smooth substrate varies much more broadly than that on the nanopillar substrate. At higher temperature, the bubble dynamics on the two substrates become even more contrasting: while the bubbles on the smooth substrate tend to merge and create either large bubbles or vapour columns starting from the substrate, those generated on the nanopillar substrate have much lesser tendency to merge and appear more uniform in size. We note that it is not likely for bubbles to coalesce after their detachment from the substrate due to liquid inertia and surface tension, thus the coalescence process mainly occurs between consecutive bubbles generated from the same nucleation site before detachment. This strongly suggests that resistance to coalescence, as well as the uniform size of bubbles generated from the nanopillar substrate result from an effective rewetting process, which facilitates bubble detachment from the surface. We hypothesise that deviation in boiling behaviours of nanopillar substrates from the smooth one, therefore, originates from the nanopillar-induced enhancement in the rewetting process.

**Figure 2:**
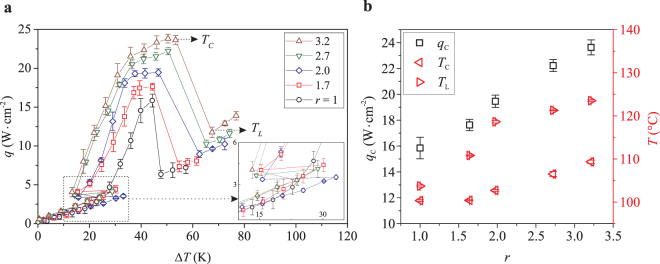
Heat flux *q* versus wall superheat Δ*T* for smooth substrate (*r* = 1) and nanopillar substrates. Error bars represent the standard deviation from multiple experiments. (**b**) Dependence of critical heat flux *q*_*c*_ (open squares), CHF temperature *T*_*c*_ (left-pointing triangles), and Leidenfrost temperature *T*_*L*_ (right-pointing triangles) on surface roughness *r*.

We now quantify the effect of nanopillars on the boiling performance by measuring the heat flux *q* for each substrate as a function of the superheat Δ*T* = *T* − *T*_*b*_, where *T*_*b*_ = 56 °C is the boiling temperature of FC-72 at atmospheric pressure. In Fig. 2a, we show the dependence of *q* on Δ*T* for the smooth and all the nanopillar substrates (See Supplementary Information for the uncertainty analysis of the heat flux and surface temperature measurements). Starting from Δ*T* = 0, *q* first gradually increases due to natural convection until it reaches the onset of nucleate boiling (Δ*T* ≈ 30 K for all tested substrates). In this so-called natural convection regime, since the nanopillars are much smaller than the thermal boundary layer thickness, their presence causes negligible effect to the heat flux, which is dictated by the convective fluid flow outside of the thermal boundary layer. Here, the boundary layer thickness is roughly 57 µm, estimated using the ratio between the natural convection heat transfer coefficient and the thermal conductivity of FC-72^24^.

As Δ*T* increases past the onset of nucleate boiling, the heat flux on nanopillar substrates, denoted as *q*^*n*^, deviates substantially from that on the smooth substrate. In particular, *q*^*n*^ after the system transitions into the nucleate boiling regime is achieved at much lower superheat compared to that when system is still in the natural convection regime (inset of Fig. 2a). For instance, for the substrate with *r* = 2, *q* = 3.3W ⋅ cm^−2^ is achieved at Δ*T* ≈ 16 K when the system is in the nucleate boiling regime, whereas a slight increase in heat flux, *q* ≈ 3.5W ⋅ cm^−2^, is achieved at much higher superheat Δ*T* ≈ 33 K in the natural convection regime. On the smooth substrate, however, the heat flux increases *smoothly* as the system transitions into the nucleate boiling regime. The presence of nanopillars causes a substantial jump in heat flux as soon as the system transitions to the nucleate boiling regime because the heat transfer mechanism is more efficient for the nanopillar substrates. The homogeneous spread of nucleation sites, high generation frequency and narrow size distribution of bubbles on nanopillar substrates (see Supplementary Information, Fig. S1) results in the sudden discontinuity in the boiling curve.

With the superheat increasing beyond the onset of nucleate boiling, bubbles are generated with higher frequency and larger size, intensifying forced convection in the bulk liquid and subsequently enhancing the heat flux *q*. For each substrate, *q* increases to the CHF *q*_*c*_ at the critical temperature *T*_*c*_, beyond which it drops sharply due to excessive vapour generation and lack of replenishing liquid to the substrate. The heat flux reaches its minimum value at the so-called Leidenfrost temperature *T*_*L*_, or the upper limit of nucleate boiling. The sudden drop in heat flux at *T*_*L*_ often triggers drastic spike in surface temperature causing the notorious “burn-out” in boiling applications. Thus, designing heat-dissipating substrates with high *T*_*L*_ is as practically important as enhancing the critical heat flux.

We observe considerable enhancement in both *q*_*c*_ and *T*_*L*_ on nanopillar substrates. In Fig. 2b, we show the dependences of *q*_*c*_ and *T*_*L*_ on the surface roughness *r*. Both *q*_*c*_ and *T*_*L*_ increase monotonically with increasing *r* (or equivalently with *l*). Compared to the smooth substrate (*r* = 1), *q*_*c*_ and *T*_*L*_ on the nanopillar substrate with *r* = 3.2 increase ≈50% and ≈25 °C, respectively. The enhancement in both *q*_*c*_ and *T*_*L*_ is a remarkable feature of the nanopillar substrates as opposed to those fabricated with larger scale structures. It was observed that micro-structures only enhance the heat flux without increasing the Leidenfrost temperature; typically, *T*_*L*_ decreases with increasing height of microstructures^25^. Separate investigations on hierarchical substrates, i.e., microstructured surfaces covered with nano-grass, reported enhancement in *either* the Leidenfrost temperature^26–28^, or the heat flux^13,27,29–34^.

### Mechanism of heat transfer enhancement on nanopillar substrates

To control the effects of nanopillars on enhancing *q*_*c*_ and *T*_*L*_, we now investigate the heat transfer mechanism on nanopillar substrates. From qualitative observations of the differences in bubble generation between the nanopillar substrates and the smooth one, we postulate that the major contributing factor leading to the observed change in *q*_*c*_ and *T*_*L*_ is the nanopillar-induced rewetting process. We note that in the case of hierarchical structured surfaces, i.e., micro-structures covered by nano-grass, enhancement in *q*_*c*_ was observed, but due to a very different mechanism: the enhancement in volume of liquid wicking through the microstructures brings more liquid to the surface and subsequently leads to an increase in heat flux by latent heat of evaporation. The wicking process, however, is not possible for nanopillars due to the tremendous viscous stress induced in flows confined in such small length scales between pillars^13^. Thus, wicking is excluded as the cause of heat flux enhancement for our nanopillar substrates, which have interspacing between pillar ≈ 360 nm.

**Figure 3:**
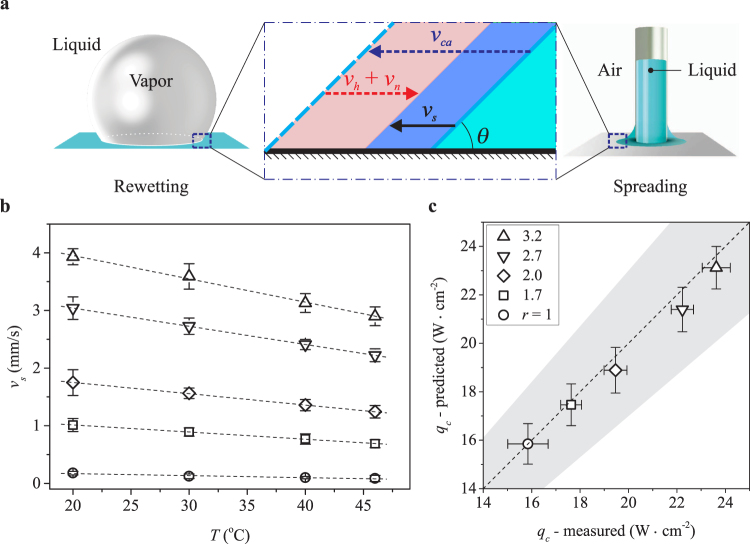
(**a**) Schematics showing similarities between the rewetting process in pool boiling (left panel) and the spreading process (right panel) from a capillary, in particular at the three-phase contact line (highlighted in the middle panel). (**b**) Apparent spreading velocity *v*_*s*_ of FC-72 on different substrates over a range of temperature from 20 °C to 46 °C. (**c**) Critical heat flux *q*_*c*_ predicted using Eqn. 2 versus the measured values for smooth substrate (*r* = 1) and nanopillar substrates. The uncertainty for the predicted heat flux comes from that of the spreading velocity measurements. The shaded area indicates a 15% deviation from the measured data.

We focus on the advancing contact line of liquid to explore how the nanopillar-induced rewetting process leads to heat transfer enhancement. Taking advantage of the similarity between the rewetting process of vapour bubbles and the spreading process of liquid (illustrated in Fig. 3a), the dynamics of the advancing contact line can be studied separately in the case of spreading liquid on nanopillar substrates^15,22^. We note that the rewetting process has been the focus of numerous detailed studies^13^, which revealed contributing factors such as surface-induced capillary force^13^, hydrostatic and hydrodynamic forces^22^, reshaping effects^35^ and vaporisation^36^. For the nanopillar substrates, we determine the dominant effects and their contribution to the heat transfer by investigating the spreading of liquid from a capillary tip onto heated substrates (see Fig. 3a). By varying the surface temperature and tracking the three-phase contact-line (TCL) as the liquid spreads, we determine the temperature dependence of the apparent spreading velocity *v*_*s*_ on each nanopillar substrate.

The surface temperature can be varied from room temperature *T*_*r*_ = 20 °C up to 46 °C without causing the liquid to boil. Although the displacement of the TCL results from various coupled effects, we simplify the analysis by decomposing *v*_*s*_ into several independent components (Fig. 3b):

1 $${v}_{s}={v}_{ca}-{v}_{n}-{v}_{h},$$

where the component *v*_*ca*_ is caused by capillary force on the substrate and therefore is a function of the surface roughness *r* only. The component *v*_*h*_ represents the reduction in spreading velocity as liquid absorbs heat from the surface and evaporates. Thus, if the substrate is not heated, i.e., *T* = *T*_*r*_, it follows that *v*_*h*_ = 0 because there is no heat transferred from the surface to the liquid. The velocity component *v*_*n*_ represents the reduction in spreading velocity due to natural evaporation of liquid. In the case of liquid spreading in air, this component is nonzero and depends on the partial pressure of vapour, whereas in the case of boiling, it vanishes as bubbles generated during boiling of liquid are filled only with saturated vapour. Here, we have neglected contributions to TCL displacement from hydrostatic pressure of the liquid column, and from capillary pressure at the upper liquid surface in the tube due to the small gap between the capillary tube and the substrate.

We postulate that the increase in heat flux on nanopillar substrates results from nanopillar-induced enhancement in wetting. This is suggested from the linear correlation between the critical heat flux of different substrates and the spreading velocity *v*_*s*_ measured on respective substrates at surface temperature *T* = 20 °C (see Supplementary Information Fig. S2). In other words, the presence of nanopillars intensifies the rewetting process, leading to an added amount of liquid brought to and subsequently evaporated from the surface. Among the contributing components to the apparent spreading velocity, *v*_*ca*_ inherits the effect of *r* on capillary wetting, thus has a direct relation to the enhanced rewetting process. In addition, as the surface temperature for spreading liquid was set lower than the liquid’s boiling temperature, we assume that with constant surface temperature, *v*_*n*_ remains fixed for all the substrates, i.e., ${v}_{n}^{n}={v}_{n}^{s}$, where the superscripts *n* and *s* respectively indicate quantities of the nanopillar substrates and the smooth one. We recall that *v*_*ca*_ is independent of *T* and *v*_*h*_(*T*_*r*_) = 0. As a result, at room temperature *T*_*r*_, the spreading velocity difference $\delta {v}_{s}({T}_{r})={v}_{s}^{n}({T}_{r})-{v}_{s}^{s}({T}_{r})$ between a nanopillar substrate and the smooth one *only* depends on the change in capillary velocity between the two substrates $\delta {v}_{ca}={v}_{ca}^{n}-{v}_{ca}^{s}$. Thus, *δv*_*ca*_ can be estimated as *δv*_*ca*_ ≈ *δv*_*s*_(*T*_*r*_).

We measure the spreading velocity *v*_*s*_ for all nanopillar substrates and the smooth substrate (see Fig. 3b). The spreading velocity increase *δv*_*s*_(*T*_*r*_) is calculated for each nanopillar substrate to obtain *δv*_*ca*_. The spreading velocity increases with increasing nanopillar height due to the increasing energetic favourability of wetted nanopillar surfaces of increasing height. The energy of the different dry nanopillar substrates is approximately the same. However, the energy of different wetted nanopillar substrates decreases with increasing height due to the larger interface between the liquid and solid nanopillar array. This energy difference drives the spreading of the FC-72, and thus, the spreading velocity increases with increasing nanopillar height. We note that for a nanopillar substrate with projected boiling area *A*_*p*_, the increase in capillary velocity *δv*_*ca*_ leads to a mass increase of evaporated liquid *ρ*_*l*_*A*_*d*_*δv*_*ca*_, where *A*_*d*_ is the time-averaged dry area during boiling on *A*_*p*_. At CHF, *A*_*d*_ can be estimated using the classic boiling model by Zuber^37^: *A*_*d*_ = *KA*_*p*_, with *K* ≈ 0.131^4,37,38^. The enhancement in heat transfer at CHF, i.e., of a nanopillar substrate compared to the smooth substrate, can be calculated as *δQ*_*c*_ = *h*_*fg*_*ρ*_*l*_*A*_*d*_*δv*_*ca*_. As a result, we can estimate the heat flux at CHF, denoted as ${q}_{c}^{n}$, through each nanopillar substrate using that through the smooth one ${q}_{c}^{s}$ and the heat flux enhancement *δQ*_*c*_/*A*_*p*_ = *Kρ*_*l*_*h*_*fg*_*δv*_*ca*_ as follows:

2 $${q}_{c}^{n}={q}_{c}^{s}+K{\rho }_{l}{h}_{fg}\delta {v}_{ca}\mathrm{.}$$

We note that the heat flux at CHF on smooth substrates depends on the surface temperature and can be predicted using the classical model for nucleate boiling by Zuber^37^. A comparative study between the model and our boiling data on the smooth substrate shows an excellent agreement (see Methods for details). Thus Eqn. 2 provides a direct prediction of the heat flux through nanopillar substrates based on the enhancement in spreading velocity. In Fig. 3c, we show a comparison between the heat flux predicted using Eqn. 2 and one measured experimentally for all the substrates. The agreement between the predicted CHF and the measured values for all nanopillar substrates strengthen our hypothesis that the enhanced heat flux is caused by nanopillar-induced enhancement in rewetting. The results also suggest that the boiling crisis is mainly dictated by the intensity of the rewetting process, and the increase in critical heat flux is a direct consequence of the faster rewetting velocity using higher nanopillars.

### Prediction of critical heat flux temperature on nanopillar substrates

We now explore how nanopillars-induced enhancement in rewetting affects the surface temperature *T*_*c*_ at CHF. Recall that the critical heat flux for nanopillar substrates can be predicted using that on the smooth substrate and the enhancement in rewetting velocity. We extend this argument to estimate *T*_*c*_ of nanopillar substrates. First, we determine the heat transfer coefficient *h*_*TCL*_ associated with liquid vaporisation at the three-phase contact line (TCL) by considering the volume Ω of liquid evaporated at TCL per unit time due to heat absorption from the nanopillar substrates. If we denote *θ* the contact angle of FC-72, Ω can be calculated as Ω = *v*_*h*_*A*_*d*_tan*θ* (see Fig. 3a for illustration). On one hand, the rate of latent heat required to evaporate Ω is *ρ*_*l*_Ω*h*_*fg*_. On the other hand, the heat rate supplied from the substrate is *h*_*TCL*_(*T* − *T*_*l*_)*A*_*d*_, where *T*_*l*_ is the liquid temperature. Balancing the rate of latent heat of evaporation and the heat rate supplied from the substrate gives the energy balance *ρ*_*l*_Ω*h*_*fg*_ = *h*_*TCL*_(*T* − *T*_*l*_)*A*_*d*_. Thus, the heat transfer coefficient *h*_*TCL*_ can be obtained by taking derivative with respect to *T* of the energy balance, assuming that *h*_*TCL*_ is independent of *T*: *h*_*TCL*_ = *ρ*_*l*_*h*_*fg*_tan*θdv*_*h*_/*dT*. Taking into account the relation between the spreading velocity *v*_*s*_ and *v*_*h*_ (Eqn. 1) and recalling that *v*_*ca*_ is independent of *T*, we have *dv*_*h*_/*dT* = *dv*_*s*_/*dT*, thus arrive at the equation to determine *h*_*TCL*_ for each substrate:

3 $${h}_{TCL}={\rho }_{l}{h}_{fg}\,\tan \,\theta \frac{d{v}_{s}}{dT}\mathrm{.}$$

**Figure 4:**
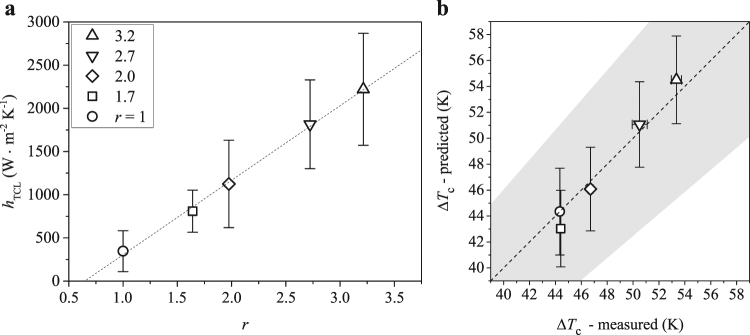
(**a**) Heat transfer coefficient at the three-phase contact line *h*_*TCL*_ versus surface roughness *r*. (**b**) Critical heat flux temperature *T*_*c*_ predicted using Eqn. 5 versus the measured values for substrates with different *r*. The shaded area indicates a 15% deviation from the measured data.

The central argument of our analysis for nanopillar-induced enhancement in heat flux is that the heat transfer increase takes place at TCL where capillary wetting and evaporation are significantly altered by the presence of nanopillars. As shown in Fig. 4a, *h*_*TCL*_ calculated using Eqn. 3 increases linearly with the surface roughness, or equivalently, the height of nanopillars. This implies that the enhancement due to both conduction at the solid-liquid interface and convection on nanopillar substrates are secondary effects. The total heat transfer coefficient *h*^*n*^ on nanopillar substrates can then be estimated using the total heat transfer coefficient *h*^*s*^ on the smooth substrate and the enhancement in heat transfer coefficient at TCL:

4 $${h}^{n}\approx {h}^{s}+K({h}_{TCL}^{n}-{h}_{TCL}^{s})+C={h}^{s}+K{\rho }_{l}{h}_{fg}\,\tan \,\theta \frac{d({v}_{s}^{n}-{v}_{s}^{s})}{dT}+C,$$

where the prefactor *K* is needed for the enhancement at TCL and the constant *C* = 425W ⋅ m^−2^ ⋅ K^−1^ accounts for the additional heat transfer caused by the flow induced by displacement of bubbles from nanopillar substrates. Since there is no obvious difference in bubble dynamics between nanopillar substrates, we use a fixed constant *C* for all nanopillar substrates to represent the heat transfer increase due to displacement of bubbles. In deriving Eqn. 4, we also assume insignificant variation in *h* between the spreading and boiling processes for a fixed substrate. We note that Eqn. 4 can be applied at CHF due to the linear dependence of *v*_*s*_ on *T* (see Fig. 3b). The heat transfer coefficient at CHF on the smooth substrate can be determined using the heat transfer balance ${q}_{c}^{s}={h}^{s}({T}_{c}^{s}-{T}_{b})$, which gives ${h}^{s}={q}_{c}^{s}/({T}_{c}^{s}-{T}_{b})$. Here ${T}_{c}^{s}$ is the wall temperature at CHF on the smooth substrate and *T*_*b*_ is the boiling temperature of the liquid. Similarly, we have ${q}_{c}^{n}={h}^{n}({T}_{c}^{n}-{T}_{b})$, where ${T}_{c}^{n}$ is the wall temperature at CHF on nanopillar substrates. Taking the predicted heat flux ${q}_{c}^{n}$ and the heat transfer coefficient *h*^*n*^ on nanopillar substrates respectively from Eq. 2 and Eq. 4, ${T}_{c}^{n}$ can be predicted as:

5 $${T}_{c}^{n}-{T}_{b}=\frac{{q}_{c}^{n}}{{h}^{n}}\mathrm{.}$$

In Fig. 4b, we compare the predicted superheat temperatures Δ*T*_*c*_ = *T*_*c*_ − *T*_*b*_ and the experimental ones at CHF for all substrates. Although the predicted values of Δ*T*_*c*_ carries the uncertainty of the spreading velocity measurement on nanopillar substrates, they fall within 15% deviation from the experimentally measured ones, highlighting a remarkable agreement between the predicted and experimental data.

## Conclusions

In summary, we show that nanopillars fabricated on boiling substrates induce a substantial effect on the boiling behaviours in the nucleate boiling regime. In particular, increasing the height of nanopillars effectively leads to considerable enhancement in both heat flux and surface temperature at CHF. We attribute such enhancement to the nanopillar-induced increase in rewetting velocity, which can be measured in a separate wetting experiment. Based on the observations that the rewetting velocity increases for substrates with higher nanopillars, as well as the assumption that the enhancement in heat flux dominantly takes place at the three-phase contact line, we develop a mechanistic model to predict both the heat flux and the temperature at CHF of nanopillar substrates. Our model takes into account the nanoscales of the pillars, and thus, excludes the wicking motion, or imbibition of fluid, as plausible mechanisms for heat transfer enhancement. As a result, the enhanced capillary force due to the presence of nanopillars is the major cause of the intensified rewetting process and subsequent increases in heat flux and temperature at CHF.

The enhancement in both of these critical quantities in pool boiling is a remarkable feature of nanopillar substrates in comparison with nano/micro-engineered substrates in which only either one can be increased. By using only nanopillars with a systematic variation in height and well-defined geometrical dimensions, we have established a direct link between the enhancement in capillary force and the boiling performance of a substrate. This provides new insights about design of surface textures not only to amplify the heat flux, but also to achieve an enhancement in the temperature at CHF, an often less discussed but equally important quantity in preventing boiling crisis.

## Methods

### Fabrication of nanopillar substrates

The nanopillar silicon substrates are obtained by inductively coupled plasma reactive ion etching (ICPRIE). The fabrication process consists of three steps, (1) manufacture of the etching mask, (2) etching the underlying silicon by ICPRIE, and (3) removal of etching mask. Polystyrene (PS) nanospheres are utilised to generate the etching mask. First, a monolayer of nanospheres with the diameter of 800 nm are self-assembled in the air-water interface^39–41^. Subsequently the monolayer is transferred to a P-type boron-doped (100) silicon substrate, which has been cleaned with acetone, methanol, isopropanol and then dried with nitrogen gas. After the substrate is dried in air at room temperature, reactive ion etching (RIE) with oxygen is applied to reduce the diameter of nanospheres to the required size (≈440 nm).

After the etching mask is manufactured, ICPRIE is used to etch the silicon substrate to fabricate silicon nanopillars with desired dimensions. During the process, SF_6_ and C_4_F_8_ with optimal ratio of 33:82 are supplied. These gases respectively serve as the etching gas and passivation gas. Different height nanopillars are obtained by varying the duration of this step. It should be noted that when the time duration is longer than 5 minutes the PS mask is impacted and the nanopillar base diameter is different from the etching mask. When the etching is completed, the PS mask is removed by ultrasonication in acetone for 5 minutes.

### Boiling experiment

The experimental setup used to measure the heat flux through different substrates consists of three sections: heating, testing and condensation. In the heating section, a cartridge heater embedded in a cylindrical copper rod is used to generate heat. The copper rod is insulated from the surrounding environment by a Teflon holder. In the testing section, a boiling substrate, which is either smooth silicon wafer or a nanopillar substrate, is placed between the copper rod and a glass cuvette of lateral size 10 mm × 10 mm. The test substrate is cleaned by ethanol and acetone in a ultrasonic bath and rinsed with distilled water before each experiment. The cuvette is filled with degassed FC-72 as a working liquid. The cuvette is enclosed by a stainless-steel container; the gap between the cuvette and the container is circulated with hot water of temperature 55.5 ± 0.5 °C to keep the temperature of the working liquid close to boiling point. The container has two glass windows on two opposite sides allowing optical recording of the boiling phenomena. A condenser is placed on top of the container to collect vapour generated from the working liquid. The cooling power of the condenser is adjusted to keep the vapour pressure inside the cuvette at atmospheric pressure.

The vapour pressure is measured by pressure transducer (Gefran) and maintained at atmospheric pressure by adjusting the cooling power of a Peltier module. The temperature is measured by K-type thermocouples and the boiling process is recorded by a high-speed camera (SA-5, Photron). Three thermocouples are distributed along the asymmetrical axis of the copper cylinder. The temperatures measured by these thermocouples are used to calculate the heat flux *q* through the test substrate and the surface temperature *T*. The heat flux in the vertical direction is approximated with the assumption that the rate of heat loss to the side is constant^43,44^. Thus the heat flux through the substrate is estimated as:

6 $$q=0.5{k}_{c}(4{T}_{2}-{T}_{1}-3{T}_{3}){\rm{\Delta }}{x}_{c}^{-1}\mathrm{.}$$

where *k*_*c*_ is the thermal conductivity of copper, *T*_1_, *T*_2_, *T*_3_ are obtained from the thermocouples in the copper rod, and Δ*x*_*c*_ = 7 mm is the distance between the thermocouples. There are three layers between the top thermocouple and the top surface: a copper layer (7 mm), a thermal glue layer (≈10 μm) and the silicon substrate (500 μm). The surface temperature *T* of the test substrate is then estimated by the one-dimensional heat conduction equation:

7 $$T={T}_{3}-q({\rm{\Delta }}{x}_{c}{{k}_{c}}^{-1}+{\rm{\Delta }}{x}_{g}{k}_{g}^{-1}+{\rm{\Delta }}{x}_{s}{k}_{s}^{-1}),$$

where Δ*x*_*c*_/*k*_*c*_, Δ*x*_*g*_/*k*_*g*_ and Δ*x*_*s*_/*k*_*s*_ are the heat resistance of copper, thermal glue, and silicon substrate respectively. For each tested substrate, the heat flux *q* and the corresponding superheat Δ*T* = *T* − *T*_*b*_ are measured when the system is in the steady state and with temperature steps of 5 K. The experimental uncertainty is obtained by repeating the experiment three times.

### Experimental Validation

**Figure 5:**
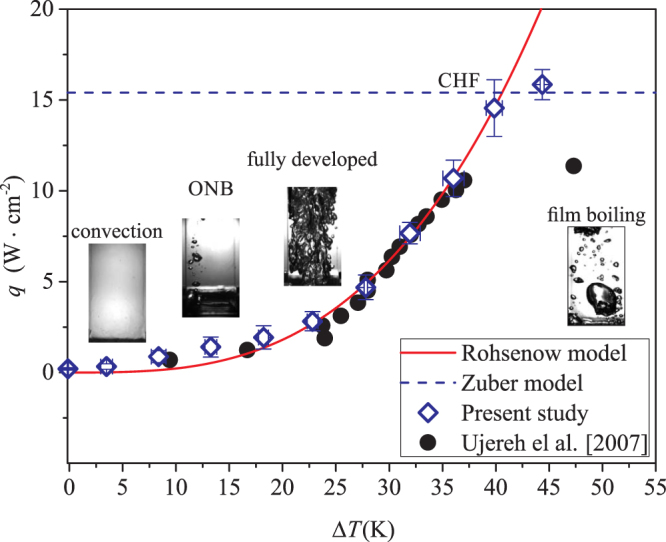
Comparison between the boiling curve measured on silicon smooth surface in the present study and the one obtained by Ujereh *et al*.^42^. The solid line represents the heat flux predicted by Rohsenow model, and the dashed line represent the critical heat flux predicted by Zuber model^37^.

The measurement procedure and analysis are validated by comparing the heat transfer data obtained on the smooth substrate to the well-known dataset obtained by Ujereh *et al*.^42^, as well as to classical model, including the one for nucleate boiling heat transfer by Rohsenow^45^ and the one for critical heat flux by Zuber^37^ (see Fig. 5). In particular, the critical heat flux on a smooth substrate is:

8 $${q}_{c}^{s}=K{\rho }_{v}^{\mathrm{1/2}}{h}_{fg}{[g\sigma ({\rho }_{l}-{\rho }_{v})]}^{\mathrm{1/4}},$$

where *K* ≈ 0.131 is an empirical parameter.

## Electronic supplementary material

Supplementary Information
