# Supplementary material for: Critical heat flux enhancement in pool boiling through increased rewetting on nanopillar array surfaces
Source: Sci Rep. 2018 Mar 19;8:4815. doi: 10.1038/s41598-018-22693-z (PMC5859279; doi:10.1038/s41598-018-22693-z)
Supplement: Supplementary file 1 — Supplementary Information [file 41598_2018_22693_MOESM1_ESM.docx]

**Critical heat flux enhancement in pool boiling through increased rewetting on nanopillar array surfaces:**

**Supplementary Information**

Thien-Binh Nguyen^1^, Dongdong Liu^1^, Md Imrul Kayes^2^, Baomin Wang^2^, Nabeel Rashin^1^, Paul W. Leu^2^, and Tuan Tran^1,*^

^1^School of Mechanical and Aerospace Engineering, Nanyang Technological University, 50 Nanyang Avenue, Singapore, 639798.

^2^Department of Industrial Engineering and Department of Mechanical Engineering and Materials Science, University of Pittsburgh, Pittsburgh, USA, 15261.

*Corresponding author: [ttran@ntu.edu.sg](mailto:ttran@ntu.edu.sg)

1. **Bubble departure diameter and releasing frequency at the onset of nucleate boiling**

**Supplementary Figure S1:** Dependence of bubble departure diameter and releasing frequency on the height of nanostructures at the onset of nucleate boiling. For the smooth surface, *l* = 0. the departure diameter tends to be smaller for structured surfaces, while there is a significant jump in bubble releasing frequency from the smooth surface to the nanostructured surfaces.

1. **Relation between the critical heat flux qc and the spreading velocity *v_s_***

**Supplementary Figure S2:** Linear relation between the critical heat flux *q_c_* and the spreading velocity *v_s_* highlighting that the spreading velocity could be used as a key parameter for predicting the critical heat flux for non-polar dielectric fluids with low surface tension, e.g., FC-72.

1. **Uncertainty analysis**

We have carried out the uncertainty analysis for the heat flux and surface temperature measurements and show the quantified values in Figure 4 below. The uncertainty analysis and the resulting quantified values for our experiment has been included in the Supplementary Information. In the revised manuscript, we also have referred to this uncertainty analysis in the Supplementary Information.

The uncertainty of the heat flux measurement comes from three main sources: thermal conductivity of copper, the distance between thermocouples, and the temperature measurement using thermocouples. The heat flux and wall temperature are calculated using the formulas:

| $q=0.5k_{c}\left( 4T_{2}-T_{1}-3T_{3} \right)\Delta x_{c}^{-1}$ | (1) |
| --- | --- |
| $T=T_{3}-q(\Delta x_{c}k_{c}^{-1}+\Delta x_{g}k_{g}^{-1}+\Delta x_{s}k_{s}^{-1})$. | (2) |

The propagated error is therefore estimated as:

| $U_{q}=\sqrt{\sum_{i=1}^{n} \left( \frac{\partial q}{\partial a_{i}}{U_{a}}_{i} \right)^{2}}$ | (3) |
| --- | --- |

where $a_{i}$ is the measured quantity *i*, and $U_{a_{i}}$ its uncertainty.

The relative uncertainty of heat flux is calculated as:

| $\frac{U_{q}}{q}=\sqrt{\frac{U_{t1}^{2}+16U_{t2}^{2}+9U_{t3}^{2}}{\left( T_{1}-4T_{2}+3T_{3} \right)^{2}}+\frac{U_{kc}^{2}}{k_{c}^{2}}+\frac{U_{x}^{2}}{x^{2}}}\times100\%$ | (4) |
| --- | --- |

where $U_{t1}$ is the uncertainty of thermocouple type K at position 1 after careful calibration:

$U_{t1}=\frac{a_{thermocouple}}{\sqrt{3}}=\frac{0.1}{\sqrt{3}}=0.06$ K

Similarly, the uncertainties of thermocouple type K at position 2 and 3 are $U_{t2}$ = $U_{t3}$= 0.06 K. The uncertainty of thermal conductivity of copper, which depends on temperature, is $U_{kc}$ = 6 W/(mK). The uncertainty of distance between thermocouples is $U_{x}$ = 0.01 mm.

The relative uncertainty of surface wall temperature can be derived using Eqn. (2) and (3) as follows:


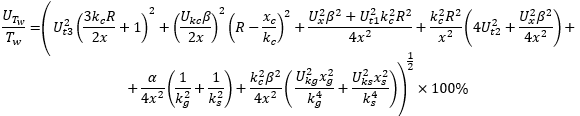
(5)

where the thermal resistance of the material layers along heat transfer direction is

$$R=\frac{x_{c}}{k_{c}}+\frac{x_{g}}{k_{g}}+\frac{x_{s}}{k_{s}}$$

The variables $\alpha$ and β respectively are

$$\alpha=U_{x}^{2}k_{c}^{2}\left( T_{1}-4T_{2}+3T_{3} \right)^{2}$$

$$\beta=T_{1}-4T_{2}+3T_{3}$$

Here, the thicknesses of the thermal glue layer and silicon substrate are $x_{g}$ = 0.1 mm and $x_{s}$= 0.5 mm, respectively. The uncertainties of thermal conductivity of thermal glue and thermal conductivity of silicon substrate are $U_{kg}$ = 0.8 W/(mK) and $U_{ks}$ = 18 W/(mK), respectively.

In Figure S3, we show all the values of relative uncertainty of all substrates at different temperatures.

**Supplementary Figure S3:** Relative uncertainty calculated for heat flux (**a**) and wall temperature (**b**) at boiling regime. At lower heat flux and superheat, relative uncertainty is below 30% and 7% respectively. As for heat flux close to CHF, relative uncertainty reduces to below 5% for heat flux and below 7% for superheat.

1. **Details on boiling experiment and set up**

The experimental setup used to measure the heat ﬂux through different substrates consists of three sections: heating, testing and condensation. In the heating section, a cartridge heater embedded in a cylindrical of copper rod is used to generate heat. The copper rod is insulated from the surrounding environment by a Teﬂon holder. In the testing section, a boiling substrate, which is either smooth silicon wafer or a nanopillar substrate, is placed between the copper rod and a glass cuvette of lateral size 10mmx10mm. The test substrate is cleaned by ethanol and acetone in an ultrasonic bath and rinsed with distilled water before each experiment. The cuvette is ﬁlled with degassed FC-72 as a working liquid. The cuvette is enclosed by a stainless-steel container; the gap between the cuvette and the container is circulated with hot water of temperature 55.5±0.5^o^C to keep the temperature of the working liquid close to boiling point. The container has two glass windows on two opposite sides allowing optical recording of the boiling phenomena. A condenser is placed on top of the container to collect vapour generated from the working liquid. The cooling power of the condenser is adjusted to keep the vapour pressure inside the cuvette at atmospheric pressure.


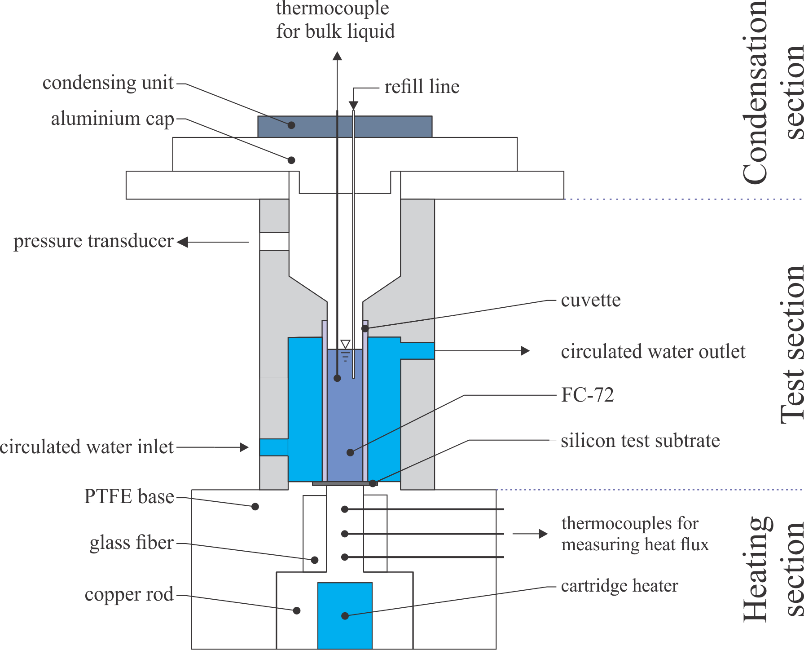


**Supplementary Figure S4:** A schematic drawing of the boiling setup. The experimental setup used to measure the heat ﬂux through different substrates consists of three sections: heating, testing and condensation.

The vapour pressure is measured by pressure transducer (Gefran) and maintained at atmospheric pressure by adjusting the cooling power of a Peltier module. The temperature is measured by K-type thermocouples and the boiling process is recorded by a high-speed camera (SA-5, Photron). Three thermocouples are distributed along the asymmetrical axis of the copper cylinder. The temperatures measured by these thermocouples are used to calculate the heat ﬂux q through the test substrate and the surface temperature T. The heat ﬂux in the vertical direction is approximated with the assumption that the rate of heat loss to the side is constant. Thus, the heat ﬂux through the substrate is estimated as:


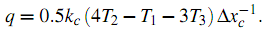


where *k*_c_ is the thermal conductivity of copper, *T*_1_, *T*_2_, *T*_3_ are obtained from the thermocouples in the copper rod, and Δ*x*_c_ is the distance between them. There are three layers between the top thermocouple and the top surface: a copper layer (7 mm), a thermal glue layer (~10μm) and the silicon substrate (500μm). The surface temperature T of the test substrate is then estimated by the one-dimensional heat conduction equation:


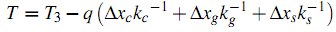


where Δ*x*_c_/*k*_c_, Δ*x*_g_/*k*_g_ and Δ*x*_s_/*k*_s_ are the heat resistance of copper, thermal glue, and silicon substrate respectively. For each tested substrate, the heat ﬂux q and the corresponding superheat Δ*T* = *T* - *T*_b_ are measured when the system is in the steady state and with temperature steps of 5K. The experimental uncertainty is obtained by repeating the experiment three times.

1. **Nanostructure sizes**

| Sample | Pillar length  *l* (nm) | Pillar base diameter  *D* (nm) | Pitch  *p* (nm) | Surface roughness *r* |
| --- | --- | --- | --- | --- |
| 1 | 260 | 440 | 800 | 1.7 |
| 2 | 390 | 440 | 800 | 2.0 |
| 3 | 690 | 440 | 800 | 2.7 |
| 4 | 1390 | 440 | 800 | 3.2 |

**Supplementary Table S1:** Size of samples in the experiment with surface roughness *r* calculated for each sample.
